# Subsidizing PGD: The Moral Case for Funding Genetic Selection

**DOI:** 10.1007/s11673-019-09932-2

**Published:** 2019-08-15

**Authors:** James M. Kemper, Christopher Gyngell, Julian Savulescu

**Affiliations:** 1 Monash Health, Clayton Road, Clayton, 3168 Australia; 2 University of Melbourne / Murdoch Children’s Research Institute, Royal Children’s Hospital, Flemington Rd, Parkville, VIC 3052 Australia; 3 Oxford Uehiro Centre for Practical Ethics, Murdoch Children’s Research Institute, Suite 8, Littlegate House St Ebbes Street, Oxford, OX1 1PT UK

**Keywords:** Preimplantation genetic diagnosis, Autonomy, Beneficence, Justice, Governmental funding

## Abstract

Preimplantation genetic diagnosis (PGD) allows the detection of genetic abnormalities in embryos produced through in vitro fertilization (IVF). Current funding models in Australia provide governmental subsidies for couples undergoing IVF, but do not extend to PGD. There are strong reasons for publicly funding PGD that follow from the moral principles of autonomy, beneficence and justice for both parents and children. We examine the objections to our proposal, specifically concerns regarding designer babies and the harm of disabled individuals, and show why these are substantially outweighed by arguments for subsidizing PGD. We argue that an acceptance of PGD is aligned with present attitudes towards procreative decision making and IVF use, and that it should therefore receive government funding.

## Introduction

### Origin

In July 2015, the biotechnology company Genea lodged an application with the Australian Department of Health to list the technology preimplantation genetic diagnosis (PGD) on the Medicare Benefits Schedule. If successful, this move would see PGD become partially or fully subsidized by the Australian federal government. This follows many public calls to improve access to PGD (Change.org 2016). Many begin like this:

In my late 20s it was discovered that I had a dominant genetic condition, this means that I have a 50% chance of passing on my condition to any children I have. After a follow up with a Genetic Counsellor I was advised that my options to prevent passing this on were the following: 1. Choose not to have children. 2. Choose to do chorionic villus sampling (CVS) early in the second trimester of a naturally conceived pregnancy and have a termination based on the result. 3. To do IVF and use pre-implantation genetic diagnosis (PGD) to screen embryos and then only use the embryos that test negative for the condition (Change.org 2017).

Option three is inaccessible to many Australians, because of its high cost; in addition to the out-of-pocket expenses associated with in vitro fertilization (IVF) (approximately A$3000–4000), PGD costs on average an additional A$6250 for an average cycle (Elhassen et al. 2015). In this paper we argue that there is a strong moral case for subsidized PGD. In section two, we outline the moral considerations in favour of subsidizing PGD. We show that there are reasons of autonomy, beneficence, and justice that support making PGD fully subsidized. In section three, we examine some of the objections to subsidizing PGD, including that it harms disabled individuals, encourages coercion, and will lead to designer babies. We argue these considerations ultimately fail and that the case in favour of subsidizing PGD is overwhelming. We begin by providing background to the technology itself.

### Preimplantation Genetic Diagnosis

First developed in 1990 (Handyside et al. 1990), preimplantation genetic diagnosis allows the detection of genetic abnormalities in embryos produced through IVF. Embryo biopsy conducted in the first six days allows deoxyribonucleic acid (DNA) to be tested for the presence or absence of specific genetic sequences. In many countries, including Australia, the United Kingdom, and Canada, PGD is available to parents whose children are at risk of inherited genetic diseases, such as cystic fibrosis or Huntington’s disease. An embryo that has been detected as containing a detrimental mutation is generally not eligible for transfer and is discarded.

PGD has been shown to be a safe and effective procedure (Chang et al. 2016) that prevents a wide range of diseases.^1^ Whilst PGD traditionally looked for specific abnormalities determined by parental analysis, modern techniques look at entire genomes for a wide array of mutations.

Currently, the Australian government, via its publicly funded healthcare system “Medicare”, provides a financial rebate of A$4800–5300 per IVF cycle (IVF Australia 2017) to couples undergoing IVF treatment. PGD is not included and, consequently, couples are required to fully finance the technology. An IVF cycle in Australia costs upwards of A$9000 (IVF Australia 2017), with PGD charged at approximately A$2500 for the first embryo biopsied and A$750 for each additional embryo (Elhassen et al. 2015). The use of genetic testing (including both PGD and preimplantation genetic screening) continues to increase in Australia, up 28.6 per cent from 2015 to 2016, being utilized in 9.16 per cent of IVF cycles (Fitzgerald et al. 2018).

## The Case for Subsidized PGD

### Autonomy

Autonomy supports the values of sovereignty and self-determination (Enoch 2017). These are particularly important principles in medicine. Patients have full sovereignty over their bodies—and their bodies can only justifiably be interfered with (be cut, injected with substances, etc.) if they validly consent to waiving those rights. Another aspect of autonomy is self-determination, which describes the rights of individuals to be the authors of their lives. Decision about weighty medical procedures, with different costs and benefits, should prima facie be left up to the individual to make in accordance with their own preferences and values.

Recently, philosophers have widened the scope of individual autonomy to include reproduction. Considerations of self-determination seem naturally to apply to decisions about procreation. Procreative decisions often have a significant impact on one’s life. If people have a prima facie right to be the author of their own lives, we ought to give parents wide scope to choose how and when they procreate, which extends to the use of reproductive technologies like PGD. The late political philosopher Ronald Dworkin was the first to describe this right to “procreative autonomy”:

The right of procreative autonomy has an important place…in Western political culture more generally. The most important feature of that culture is a belief [in] individual human dignity: that people have the moral right—and the moral responsibility—to confront the most fundamental questions about the meaning and value of their own lives for themselves, answering to their own consciences and convictions … The principle of procreative autonomy, in a broad sense, is embedded in any genuinely democratic culture. (Dworkin 2011)

The idea of procreative autonomy has been further developed in the work of John Harris (2000) and John Robertson (2003).

Having a child with a serious disability would significantly affect how one’s life would go. A concern for self-determination suggests people who are having a child should, as much as possible, be free to influence whether or not that child has a disability. Currently the cost of PGD is a barrier to this freedom. Considerations of autonomy therefore favour making PGD publicly funded.

This line of thinking brings up considerations of the limits of parental autonomy. What if parents want a child with certain characteristics, including a disability; should they be free to make this choice? Such considerations have been discussed at length elsewhere (Harris 2010). We will put this issue aside for now, taking the position that in such cases consideration of parental autonomy does in fact count in favour of free parental choice. However, in many controversial cases, there are other factors which count against autonomy (harm to the child, harm to society) that are absent in the use of PGD to prevent disease. Even the staunchest critics of genetic selection (Habermas 2003; Sandel 2004) are in favour of selection against diseases.

### Beneficence

Beneficence is one of the foundations on which the practices of both parenthood and medicine are established. The key notion behind beneficence is that of benefit. Beneficence demands parents and professionals act in the best interests of their children and patients.

#### Parents

As discussed in section 2.1, were PGD to be publicly funded, parents would benefit by having more reproductive options. The earliest diagnostic confirmation of genetic abnormalities currently available via antenatal testing is at or beyond eleven weeks’ gestation. At this stage the mother has carried her child for over two months and may have created a psychological and emotional bond. Were a couple to be informed that their fetus had some form of abnormality, abortion would be a much harder decision to make, and the procedure more invasive. Compare this to PGD, which can provide a diagnosis prior to the embryo being transferred into the mother’s uterus; a decision to discard the embryo at this stage should, theoretically, be much easier. Furthermore, a greater percentage of individuals would believe a two or three-month old fetus is a moral person than would believe the same of a five-day-old embryo.

#### Children

Imagine a couple—Jane and Jay—are both carriers of a mutation that causes spinal muscular atrophy (SMA), a genetic condition that normally causes death in the first two years of life. Jane and Jay undergo IVF and PGD and have a healthy child, Del.

Has Del benefited from Jane and Jay’s decision to undergo PGD? On some views of benefit, an action can only benefit someone “if it makes her better off in some respect than she would have been, had the action not been performed” (Gardner 2015, p329).^2^ Imagine if Jane and Jay do not conceive through IVF. Does this leave Del in a worse state? No, rather Del will not exist at all. On this view, Del doesn’t benefit from the action of her parents—for there is no scenario that Del exists and has SMA.

However, there are different ways to understand the requirements of beneficence (Gardner 2015). We could concede that PGD does not benefit Del, but rather benefits Jane and Jay’s “next child”—whoever that particular person may be (Parfit 2017). The relevant comparison to make is between Del’s well-being and the well-being of the child Jane and Jerry would have had, had they not engaged in PGD. In cases where that next child would have had SMA, PGD confers a significant benefit.

This is another way of saying that PGD confers an impersonal, rather than a person-affecting benefit. That we should understand beneficence in such an impersonal way has long been argued in bioethics. Savulescu’s (2001, p415) principle of *procreative beneficence* states “couples (or single reproducers) should select the child, of the possible children they could have, who is expected to have the best life, or at least as good a life as the others, based on the relevant, available information*.*” This appeals to the idea that parents have obligations to benefit their children in general—regardless of their identity.

Take the example of the Zika virus. If a woman falls pregnant during a Zika infection, it is highly likely the baby will be born with microcephaly. During the Zika outbreak, many health systems recommended that women who had visited Zika affected areas wait at least three months to conceive. In doing so, they were effectively recommending that women avoid the birth of infants predisposed to disability and instead aim to conceive clinically “normal” infants. Such advice supports procreative beneficence. Indeed, society also encourages couples to select the appropriate timing to have a child in relation to other factors: finances, careers, housing, relationship stability, and parental age. This reflects that we generally do not understand benefit in a narrow person-affecting sense

If we understand beneficence in a broad, impersonal way, then it is clear beneficence strongly favours making PGD widely available.

### Justice

#### The Basis of PGD Justice

The Roman lawyer Cicero gives one of the earliest definitions of justice as “the virtue which assigns to each his due” (Cicero 1933, p321). This broad definition still captures the core concerns of justice today. While justice encompasses many elements of ethics and law—such as the punishment of crimes, the distribution of resources, and the relationship between society and individuals—it fundamentally represents a concern for giving people what they are “due.” In moral philosophy, justice is seen as a particularly powerful value. If something is labelled as “unjust”—be it an institution, policy or individual action—this generates a strong, if not decisive, reason to reject it (Miller 2017).

The medical resources required for PGD—drugs, a specialist’s time, and so on— are limited. The demand for these resources outstrips their availability. This raises the question of how to allocate them in a way that “assigns each their due.”

The current funding arrangement, whereby IVF is subsidized but PGD services receive no rebate via Medicare, ensures PGD continues to be available only to relatively well-off individuals. Access and utilization of IVF services is greater in countries with a higher gross domestic product per capita (Zegers-Hochschild 2006), and it has been noted that only in countries with minimal out-of-pocket expenses (i.e. significant and sufficient government subsidies) does the utilization of IVF services approximate demand (Adamson 2009). A comparison can be made to the healthcare funding arrangement seen in the United States of America, where there is no national universal healthcare system; currently, PGD receives no governmental support or subsidies, thereby ensuring it remains segregated from lower socioeconomic couples.

If PGD were to become subsidized, would this promote justice? There are several different theories of distributive justice which give different answers to the question of how we should distribute a limited resource. Strict egalitarianism holds that each member of society receives exactly the same proportion of the distributed goods. John Rawls “maximin” theory holds that inequalities are morally permissible as long as they improve the level of the least advantaged in society (Rawls, 1971) “Maximin” is a type of theory of prioritarianism—a group of theories which argue that when distributing scant resources, priority should be given to those who are the worst off.

Another version of prioritarianism is “sufficientarianism,” according to which priority is to be given to the worst off, but only when they are below some minimum threshold. In “Rights, Utility and Universalization,” John Mackie defends a version of sufficientarianism under which everyone has a right to a fair go (1984). According to a maximizing version of giving people a “fair go,” we should give as many people as possible a decent chance of having a good life. This is a plausible, common-sense principle of justice. A fair go entails that each person has a legitimate claim to a sufficient quality of life. If everyone has a sufficient level of well-being, distribution should be performed according to a utilitarian principle of maximizing benefits. According to the Right to a Fair Go principle, or sufficientarianism generally, medical resources should be distributed to maximize the number of people that have a sufficient quality of life. If two distributions both result in the same number of people having a sufficient quality of life, whichever one maximizes overall well-being should be preferred.

All interpretations can be utilized in arguing a case for the public funding of PGD services. It is desirable to have access to PGD services as some people want to avoid their children having serious disabilities. Currently, however, financial barriers mean that only the wealthy have access to it. Given the impact an unwell or disabled child can have on the financial status of a family, the argument for taxpayer funding of PGD is strengthened amongst low socioeconomic families. A lack of access to PGD could make people even less well-off and drop them below the minimum threshold for having a fair go.

Most importantly, justice requires making PGD free in the following way. All resources, including healthcare resources, are limited. Justice requires that these be used to bring about as much benefit to people as possible (utilitarianism) or to bring as many people above a sufficient threshold (sufficientarianism). The healthcare of people with genetic disorders is significant. By avoiding genetic disorders, these resources are made available to other people requiring healthcare. These resources could be significant, as we will now argue, and thus justice requires making PGD free.

#### Health Systems

The growing health expenditure experienced by many countries, with the direct pressure on the government’s health budget, delivers an ongoing challenge with regards to the ability to fund all healthcare demands. The public funding of PGD may seem like an additional burden on these limited funds. The Australian Government’s Medical Services Advisory Committee (MSAC), tasked with reviewing Genea’s application, notes that the financial estimates of the net cost to the budget for subsidizing PGD to be A$3.9 million in year one and A$6.8 million in year five (Medical Services Advisory Committee 2017), a not-insignificant value.

However, while publicly funding PGD will represent a significant upfront cost for health systems, these costs will be recouped in the long term. By reducing the frequency of deleterious genetic conditions, PGD will decrease the demand placed on the health budget by such diseases. The increased availability of funding can be utilized in other sectors of healthcare, with the aim of further improving the justice and equality of all members of the community.

As part of Genea’s submission to MSAC, they were asked to provide an economic evaluation of subsidizing PGD. The incremental cost per unaffected live birth following PGD was calculated as A$32,727 (taking into account both the cost of PGD and the success rate of IVF); however, this is offset by the mean lifetime health system cost of many diseases (for example, A$335,000 for an individual with cystic fibrosis), signifying a significant saving in healthcare expenditure (Medical Services Advisory Committee 2017). Whilst the data from this one condition may not be easily extrapolated to incorporate genetic predispositions for conditions such as cancer, at its least it serves to underline the call for PGD to be publicly funded for certain genetic abnormalities.

As many genetic mutations demand increased healthcare access in the first years of life, some of the financial benefits of publicly funding PGD should be quickly realized. We hypothesize that the harm produced by redistributing funds towards subsidizing PGD (and thus depriving other areas of the budget of this money) will be minor compared to the significant benefit a reduction in genetic diseases can provide to the healthcare budget in the long term. Indeed, MSAC acknowledge that “PGD was likely to be cost-effective or cost-saving … in conditions with high lifetime costs,” and went on to recommend that PGD be publicly funded in Australia (Medical Services Advisory Committee 2017, 2). This argument is akin to the aim of preventative medicine: addressing causes of ill health prior to the development of symptoms (at a cost to the current healthcare budget) and thereby reducing future costs. Governments are all too often accused of short-term, electoral-cycle thinking; the short-term harms of subsidizing PGD should be acknowledged but set aside in favour of the long-term benefits.

As an example of another condition with a strong case for subsidized testing through PGD, take Gaucher's disease (Gyngell and Savulescu. 2018). It is caused by a genetic defect which results in the reduction or absence of the enzyme glucocerebrosidase. This can damage the liver, spleen, lungs, and kidneys, and can be lethal. Fortunately, there is an effective treatment. A modified version of the enzyme can be produced in the laboratory and administered directly into a patient’s blood stream.

Many will say that as we already have a treatment for diseases like Gaucher’s disease, we do not need genetic selection. However, the cost of producing the replacement enzyme is very high, with the total cost of treating each patient with Gaucher's disease ranging between US$200,000 and US$400,000 per year per person (van Dussen et al. 2014). Over a patient’s lifetime the cost is approximately US$9 million. This is many times above the cost effectiveness threshold used by many public health systems, but in many cases it is still covered as an “orphan” drug.

In public health systems with limited resources, an expensive therapy has the opportunity cost of preventing the treatment of someone else's disease. Justice requires we choose the most cost-effective option. Genetic selection could potentially prevent Gaucher's disease in one cycle. The cost would possibly be in the range A$10,0000 in total per person, compared with A$9 million lifetime of treatment through enzyme replacement. IVF and PGD would increase the efficiency of public health systems and allow more diseases to be prevented. This would disproportionately benefit those in lower socio-economic groups who rely more on public health systems. This is another way subsidizing PGD can remedy existing injustices.

## Objections

In this section, we examine some of the possible arguments against making PGD publicly funded. We argue these objections can be mitigated or disregarded.

### Additional IVF Cycles

One concern occasionally raised is that subsidizing PGD would lead to an increase in the number of IVF cycles undertaken by patients. We are not advocating for the exclusive use of IVF services in preference to natural conception; the public funding of PGD is to be used for couples already requiring or utilizing IVF. Furthermore, irrespective of the use of PGD, selection of embryos is still conducted by various techniques, most commonly based on the morphological appearance and characteristics of embryos. PGD seeks to improve the selection process, by categorically grading an embryo based on genetic normality. By selecting “superior” embryos, PGD is likely to improve the chance of pregnancy success, as poor-quality embryos have an increased chance of uterine transfer failure or miscarriage; the improved time to live birth rates will decrease the requirement for additional IVF cycles to achieve a live birth. Given the significant out-of-pocket expense of accessing IVF services, it is unlikely that publicly funding PGD will cause a marked increase in couples abandoning natural conception techniques to utilize IVF. Even if free PGD were to increase the number of IVF cycles, this would be unproblematic as long as couples were adequately informed of the risks and overall the procedure remained cost-effective relative to fewer IVF cycles.

### Moral Status of Embryos

Discarding embryos based on their genetic composition has caused concern amongst some authors (Kass 2003; Sandel 2004). They feel that to reject an embryo is to reject a child. Numerous discussions have focused on the debate regarding at which stage of development a group of cells can be classified as human and therefore have some or all human rights assigned. One extreme version of this view holds that an embryo enjoys full moral protection against destruction.

This obviously prohibits PGD entirely, but also raises ethical doubts about IVF and other commonly accepted practices.

In those jurisdictions where multiple embryos are produced as a part of IVF, one must conclude that an embryo is not yet a person. IVF typically seeks to harvest multiple oocytes, some of which may never be used; some will fail fertilization, some embryos will never develop sufficiently to be transferred, and others may be surplus to requirements. One must therefore accept that disposal of embryos is a component of IVF treatment. Those that have accepted this but continue to oppose the selection of embryos based on certain traits should be reminded of the IVF process. Embryos with low morphological (developmental) ratings are often removed as they have an increased likelihood of failure. Therefore, all embryos undergo some form of selection based on their traits, be they morphological, developmental, or in the case of PGD, genetic.

In some jurisdictions, such as Germany, only one embryo is produced. There it would be consistent to reject PGD (as Germans do). But where PGD is acceptable, it should be publicly subsidized because of its significant benefits and for reasons of distributive justice.

It must also be recognized that other accepted practices also increase the number of embryos not carried to term, most notably contraceptive measures. Within most countries, contraception is a widely established and advocated for notion. It should therefore be accepted that for most individuals in a democratic society, the moral status of an embryo does not prevent the use and public funding of PGD.

### Designer Babies

One of the premier concerns elicited when discussing the selection of genes is the notion of “designer babies”—babies chosen for particular characteristics. These concerns are often linked to worries over eugenics, and specifically the Nazi campaign to breed blue-eyed, blonde-haired children (Appel 2012). As Leslie Whestine writes “designer babies and genetic engineering … provoke our collective anxieties more than in any other scientific discipline” (2015, p547).

This “slippery slope” argument suggests that PGD will soon be involved in selecting aesthetic qualities which have no impact on health and well-being. It must be recognized that the current legal framework surrounding PGD in Australia, the United Kingdom, and other jurisdictions regulating IVF does not allow such selection and that unless society allows governments to alter these laws, PGD will never be used for such purposes. The argument we are making in this paper refers to the public funding of *already-approved* PGD services; there is to be no alteration to the accepted conditions, simply an amendment to PGD’s financial funding. In its report, the MSAC discusses the implementation of a “gate-keeper function,” to ensure the use of publicly funded PGD services only for conditions with an “acceptable evidence of clinical benefit and cost-effectiveness” (Medical Services Advisory Committee 2017, 3). We are strong advocates for the continued role of and supervision by oversight committees, such as Australia’s MSAC or the United Kingdom’s Human Fertilisation and Embryology Authority, whose responsibility it will be to ensure PGD is only used for approved medical conditions. Therefore, whilst the notion of “designer babies” is commonly used by the media to evoke an emotional response from audiences, if society holds governments accountable for any changes to PGD laws, it is unlikely that PGD will be used in such a manner.

### Harm to Disabled People

Some authors have argued that to prevent the transfer of an embryo destined to be disabled is to label the lives of all disabled individuals as worthless. Helen Watt states that any attempt by PGD to limit the natural creation of disabled embryos is to “send out a highly demoralizing message to born disabled people” (2004, p57 ). This, sometimes called the expressivist argument (Hofmann 2017), puts the case that subsidizing PGD would increase discrimination against those with disabilities.

There is a fear that reaffirming these genetic disorders as not “normal” will strengthen prejudice towards disabled individuals. This should not be the case, and with appropriate education and support, PGD can be used to reduce the incidence of such congenital disabilities whilst still supporting individuals and families to live meaningful and fulfilling lives. As Watt (2004, p57) herself notes, disabled individuals “are limited in certain respects,” and we doubt most patients would wish their medical condition or disability on a future child. The fact that “having a child with disabilities can be of great value to the parents” (Watt 2004, p59) is undoubtedly true. It is unlikely, however, that the majority of parents fall pregnant with the desire to have a disabled child. This is the value of PGD: it seeks to prevent future cases, not to undermine the care and treatment of current ones. Indeed, MSAC’s report to the government clearly notes “that the purpose of PGD testing is not to reduce the number of individuals deemed costly to society, nor to degrade society’s willingness to care for those born with a genetic abnormality” (Medical Services Advisory Committee 2017, 1). Society strives to find a cure for cancer, not to inform cancer patients that they are insignificant, but rather to prevent future cases from ever developing. As Petersen writes, “slippery slope arguments against PGD tend to focus only on possible harms to disabled persons … [ignoring] the increase in welfare of other individuals that might be gained by PGD” (2005, p233).

Another way in which subsidizing PGD could negatively impact disabled individuals is by reducing the total number of disabled people. Consider people who use wheelchairs: whilst the prevalence is high, governments and societies have a strong impetus to ensure buildings have wheelchair ramps and appropriate access services. As the prevalence of such individuals decreases, however, the concern is that society will no longer justify the additional requirements (financial, spatial, etc.) required to facilitate wheelchairs. However, for other disabilities, the public’s moral incentive to assist disabled individuals will remain unchanged; if this is the case, services should continue to be provided as needed. Indeed, services may also improve proportionally to the decreased incidence of disabilities; Gyngell and Douglas (2018) consider the case of in-home nursing care: with a reduction in demand, the committed financial expenditure will increase per person, thereby allowing improved services and access. Furthermore, humans will continue to have accidents and diseases which render them, in this case, unable to mobilize without a wheelchair, thereby ensuring appropriate services continue to be delivered. PGD thus should not necessarily be seen as a threat to disabled individuals, as long as society retains its moral incentive to care for people living with disabilities.

Juth expresses concern that positive selection against disabilities and other medical conditions will create societal bias in which “having a disabled child is gradually made so unattractive that the use of PGD becomes the only bearable choice for many couples” (2012, p5). It must be acknowledged that almost every technology has the potential to create poor consequences, but that this is not a reason to abandon the technology (Petersen 2005). We believe measures can be undertaken to prevent this from occurring, should society find itself heading down this path. Firstly, even compulsory IVF and PGD would not entirely eliminate disability, there will still be disabled children through accidents, in utero exposure to toxins, and so on. Secondly, as we have previously stated, for the well-being of the future child, we should want and encourage parents to undergo PGD to reduce the incidence of disability.

### Coercion

Concerns have been raised regarding the impact of publicly funding PGD services with regards to strength of the social and moral obligations it places on aspiring parents and the opportunity for coercion.

Watt states that good parenthood requires “unconditional acceptance” and that “parents who make their love and care conditional on their child displaying certain features [can be] seen as unparental” (2004, p52). We feel a distinction must be made; parents should accept their offspring when events beyond their control occur: for example, a child who becomes disabled following a car accident or one who develops cancer. However, if the parents have the ability to influence the outcome and they choose not to, then they should be held morally accountable for their inaction. In the context of disability or cancer, the public would not excuse a parent who failed to prevent their offspring getting into an accident or being exposed to toxic material. They may even be criminally liable. A society that has the opportunity to test for disability or a cancer predisposition but fails to do so due to a lack of funding should bear some responsibility for the suffering caused. Savulescu and Kahane note that “most people will agree that there is a moral defect in parents who intend to conceive a child but are indifferent to whether their future child will be born with the potential for a good life” (2009, p276). Returning to Feinberg’s concept, by not testing for genetic abnormalities, parents may limit the future possibilities experienced by their offspring (1980). Parents “have a duty to perform [an action such as PGD] with a high degree of care and in the best interest of the resulting child” (Malek and Daar 2012, p8).

Nonetheless, we might think that public funding for PGD might increase the tendency for coercion. However, it is important for society to remember that coercion is inappropriate and is unacceptable in other areas of healthcare. Public funding of immunization programs and prenatal trisomy screening provide similar scenarios to subsidizing PGD. We do not think it is inappropriate to subsidize immunization just because of the potential for coercion. Coercion should be resisted but that is a separate issue.

Moreover, it is not obvious that coercion is always wrong. Laws are coercive. Malek and Daar (2012, p7) neatly describe the legal aspects of PGD technology . They comment that parents who undertake IVF may be legally liable should they refuse to undergo PGD “in the context of its harm-preventing benefit.” This would be especially applicable to couples with a known risk of transmitting a genetic condition. The question then arises as to which party pursues legal action—can a child, limited by illness or disability, seek legal retribution for their parents’ inaction? Is the physician or even the state liable for failing to impose mandatory PGD testing in such circumstances? We would suggest one solution is to make PGD “free” for IVF patients; this allows all parents to access PGD services and is likely to reduce the proportion of patients not undergoing PGD testing. One could argue parents are still able to refuse this opportunity: this could only be negated by mandating IVF patients undergo PGD. Malek and Daar (2012, p7) suggest current laws clearly require parents “to act (for example, by providing necessary food and shelter) and to refrain from acting (by not intentionally placing a child in harm’s way).” They predict that as the laws are refined in relation to IVF, a parental obligation to “maximize the well-being of their future offspring by all reasonable means” (Malek and Daar 2012, p7) will be imposed on parents, thereby almost mandating the use of PGD. Whilst parental autonomy is a highly regarded legal status, most Western legal systems will disregard this principle to maintain the best interests of the child. Consider for example the regularly cited cases whereby Jehovah’s Witness parents refuse life-saving blood transfusions for their child; in the majority of circumstances, “the child’s welfare supersedes personal freedoms” (Malek and Daar 2012, 7). Adam Zimmerman (2009) writes that such laws are strictly utilitarian, in which the future well-being of the child, and hence society, surpass any one individual’s agency. The purposes of this paper is not to investigate the framework surrounding IVF and laws regarding embryos, fetuses, and children; for a summary discussion in the context of the American legal system, we refer readers to Malek and Daar (2012). However, the above discussion highlights the benefit taxpayer funding of PGD services would bring to any legal adjudication; were PGD to be widely and freely available, this would remove significant potential justifications for parents choosing not to undergo testing. Whilst these legal precedents may again raise concerns of coercion, a decision must be reached on whether a risk of coercion is acceptable to allow PGD to benefit significant numbers of future children.

## Conclusion

Throughout this article, we have highlighted the numerous justifications for the widespread implementation of PGD for the majority, if not all, IVF pregnancies. One of the easiest ways to facilitate such a proposal would be to publicly fund PGD to achieve access equality for all couples. If one agrees embryo selection is an ethically and morally sound practice, then PGD enables parents to fulfil their moral duty and personal desire to choose the best child available to them. The future impact on the broader community of producing healthier offspring will extend far beyond the financial savings on health expenditure; this will only broaden as further genetic discoveries are made. Society has a moral obligation to utilize technologies such as PGD to reduce disability and disease, and it should be publicly funded.

In those jurisdictions where PGD is already legal and available, there are overwhelming arguments to subsidize. Indeed, there are no good objections against it. If PGD is made available in IVF, it ought to be free.

## Footnotes

1 For a comprehensive list, see http://guide.hfea.gov.uk/pgd/.

2 This view is articulated, but not endorsed, by Gardner.
